# Celiac Disease-Type Tissue Transglutaminase Autoantibody Deposits in Kidney Biopsies of Patients with IgA Nephropathy

**DOI:** 10.3390/nu13051594

**Published:** 2021-05-11

**Authors:** Rakel Nurmi, Ilma Korponay-Szabó, Kaija Laurila, Heini Huhtala, Onni Niemelä, Jukka Mustonen, Satu Mäkelä, Katri Kaukinen, Katri Lindfors

**Affiliations:** 1 Celiac Disease Research Center, Faculty of Medicine and Health Technology, Tampere University, 33520 Tampere, Finland; rakel.nurmi@fimnet.fi (R.N.); ilma.korponay-szabo@tuni.fi (I.K.-S.); kaija.laurila@tuni.fi (K.L.); katri.kaukinen@tuni.fi (K.K.); 2 Celiac Disease Center, Heim Pál National Pediatric Institute, 1089 Budapest, Hungary; 3 Department of Pediatrics, Faculty of Medicine and Clinical Center, University of Debrecen, 4032 Debrecen, Hungary; 4 Faculty of Social Sciences, Tampere University, 33520 Tampere, Finland; heini.huhtala@tuni.fi; 5 Medical Research Unit, Seinäjoki Central Hospital, 60220 Seinäjoki, Finland; onni.niemela@epshp.fi; 6 Faculty of Medicine and Health Technology, Tampere University, 33520 Tampere, Finland; jukka.mustonen@tuni.fi (J.M.); satu.m.makela@pshp.fi (S.M.); 7 Department of Internal Medicine, Tampere University Hospital, 33521 Tampere, Finland

**Keywords:** IgA nephropathy, celiac disease, tissue transglutaminase autoantibody, tissue transglutaminase-targeted IgA deposits

## Abstract

An association between celiac disease and IgA nephropathy (IgAN) has been suggested. In celiac disease, in addition to circulating in serum, IgA-class tissue transglutaminase (tTG) autoantibodies are deposited in the small bowel mucosa and extraintestinal organs. In this case series of IgAN patients with or without celiac disease, we studied whether celiac disease-type IgA-tTG deposits occur in kidney biopsies. The study included nine IgAN patients, four of them with celiac disease. At the time of the diagnostic kidney biopsy serum tTG autoantibodies were measured and colocalization of IgA and tTG was investigated in the frozen kidney biopsies. Three IgAN patients with celiac disease had IgA-tTG deposits in the kidney even though in two of these the celiac disease diagnosis had been set years later. These deposits were not found in a patient with already diagnosed celiac disease following a gluten-free diet. Of the five non-celiac IgAN patients, three had IgA-tTG deposits in the kidney. We conclude that tTG-targeted IgA deposits can be found in the kidney biopsies of gluten-consuming IgAN patients but their specificity to celiac disease seems limited.

## 1. Introduction

Celiac disease, an immune-mediated enteropathy, is driven by the ingestion of cereals, wheat, rye, and barley containing gluten and characterized by a disease-specific autoantibody response targeting tissue transglutaminase (tTG) [1]. During gluten consumption, IgA-class tTG autoantibodies circulate in serum but are also deposited in the small bowel mucosa, where they are bound to their antigen tTG around mucosal capillaries and on the basement membrane below the mucosal epithelium [2,3]. Interestingly, these small intestinal IgA deposits may be present even before the development of small bowel mucosal villous atrophy or the detection of the tTG autoantibodies in the circulation, and they may predict forthcoming manifest celiac disease [3,4,5]. Moreover, IgA deposits in the gut have also been found in celiac disease patients with negative serum tTG autoantibodies [6,7]. Upon introduction of a gluten-free diet (GFD), the gold standard treatment for celiac disease, these small intestinal mucosal deposits disappear along with serum tTG autoantibodies [4,8]. IgA-class tTG-targeted autoantibody deposits can be also found in several other tissues, including liver, muscle, and brain, often coinciding with extraintestinal manifestations of celiac disease affecting the organ in question (e.g., hepatitis, muscle weakness, and ataxia) [3,7]. However, little is known about the occurrence of such extraintestinal IgA-tTG deposits long before diagnosis of celiac disease or their dependence on the presence of gluten in the diet.

It has been proposed that celiac disease may be associated with IgA nephropathy (IgAN), globally the most common primary chronic glomerular disease [9,10]. The diagnostic hallmark of IgAN is the predominance of hypo-galactosylated IgA1 deposits in the mesangium of the glomeruli [11]. In IgAN, the interaction between environmental antigens, dysregulation of IgA immune responses and pathogenic circulating IgA complexes eventually leads to IgA1 deposits in the kidney [12]. Although celiac disease and IgAN target different organs, they have a great deal in common. As mentioned above, aberrant IgA response is involved in both diseases [13]. tTG is known to play a decisive role in the pathogenesis of celiac disease by modifying wheat gluten-derived gliadin into a more immunogenic form [14]. Although the role of tTG in IgAN is less clear, studies conducted in both human patients and mice suggest that tTG is needed for the development of IgAN-type mesangial IgA deposits and the impairment of the clinical course of IgAN [15,16]. The ingestion of dietary gluten is required for the development of celiac disease, and may also promote the development of IgAN, at least in mice [14,17,18]. In addition, GFD being the only treatment for celiac disease, it is intriguing that some reports have described clinical improvement of IgAN with the same diet [19,20,21]. 

The association between celiac disease and IgAN is still being actively researched. In this case series we investigated whether celiac disease-type tTG-targeted IgA autoantibody deposits occur in the kidney biopsies of IgAN patients with or without concomitant or subsequent celiac disease.

## 2. Materials and Methods

### 2.1. Patients and Clinical Data

This retrospective study reports a case series of nine adult IgAN patients on whom diagnostic kidney biopsy was performed during the period 1981–1987 at Tampere University Hospital, Finland. IgAN was defined as glomerulonephritis with typical light microscopy features and IgA as the sole or main glomerular immunofluorescence finding [9]. Four of the IgAN patients also had diagnosed celiac disease. Five non-celiac patients who had received their IgAN diagnoses during the same period were selected as controls. The patients were followed-up until recent available laboratory results or death. Clinical data at the time of the IgAN diagnoses and at follow-up were collected from the medical records and included demographic data, creatinine values, and data on celiac disease diagnosis. The outcomes (chronic dialysis, kidney transplant, and death) were documented. Creatinine values and the CKD-EPI (Chronic Kidney Disease Epidemiology Collaboration) equation were used to determine estimated glomerular filtration rate (eGFR). eGFR ≥90 indicates normal kidney function, eGFR 30–59 moderate renal impairment, eGFR 15–29 severe renal impairment, and eGFR <15 renal failure [22]. 

### 2.2. Determination of Serum and Tissue tTG-Targeted IgA Autoantibodies 

Stored frozen (−80 °C) serum samples taken at the time of kidney biopsy were used to identify IgA-class tTG autoantibodies using the ELIA Celikey assay (Celikey^®^, Phadia, GmbH, Freiburg, Germany) according to manufacturers’ instructions. Values higher than 7.0 U/mL were regarded as positive.

Celiac disease-type tissue deposits with colocalization of IgA and tTG were determined in frozen biopsies by evaluators blinded to the clinical data. These included kidney biopsies for diagnosing IgAN and small bowel mucosal biopsies of two celiac disease patients taken at the time of the celiac disease diagnoses. The deposits were detected using the technique described earlier by Korponay-Szabó et al. [3] from snap frozen biopsies embedded in optimal cutting temperature compound (OCT, Tissue Tek, Sakura Finetek Europe B.V., AJ Alphen aan den Rijn, The Netherlands). The frozen sections of 5 µm thickness were stained with fluorescein isothiocyanate (FITC)-labeled rabbit antibodies against human IgA (Dako AS, Glostrup, Denmark) at a dilution of 1:40 in phosphate buffered saline and with monoclonal mouse antibodies against tTG (CUB7402, NeoMarkers, Fremont, CA, USA), which were detected with rhodamine-conjugated anti-mouse immunoglobulin rabbit antibodies (Dako) diluted 1:200 in phosphate buffered saline.

The kidney samples were investigated for the presence of tTG and IgA. IgA deposits around the basement membrane of extraglomerular blood vessels, of the parietal layer of Bowman’s capsules and of the proximal or distal tubuli in colocalization with tTG were regarded as celiac disease-type deposits. Although the glomerular capillaries also contain tTG around their basement membrane, IgA deposition on tTG within the glomeruli was not clearly discernible because all patients had extensive mesangial IgA deposits related to IgAN itself. Therefore, glomerular IgA was not taken into account for celiac disease-type deposit evaluation. 

In the small bowel mucosal biopsies, subepithelial IgA deposits found on the basement membrane below the villous and crypt epithelium and around the mucosal blood vessels were regarded as celiac disease-type IgA deposits, as in non-celiac subjects small-bowel mucosa IgA is detected only inside the plasma and epithelial cells [3,6]. Colocalization of IgA and tTG was regarded as celiac disease-type deposits. 

### 2.3. Ethical Considerations

Informed consent for the study was obtained from the patients. The research protocol (E99105, R20056) was approved by the Regional Ethics Committee of Pirkanmaa Hospital District. The study protocol follows the ethical guidelines of the Declaration of Helsinki. 

## 3. Results

**Figure 1:**
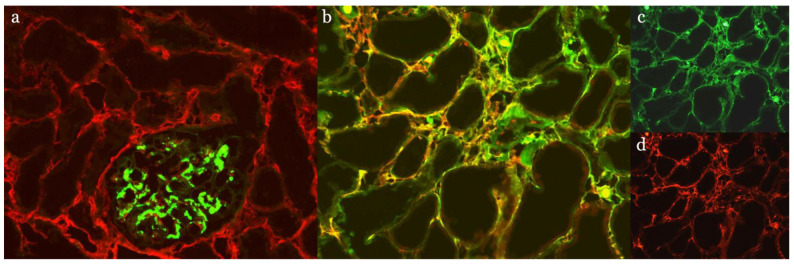
Immunofluorescent staining for immunoglobulin A (IgA) (green) and tissue transglutaminase (tTG) (red), and their colocalization (yellow) in the renal biopsies taken at the time of the IgA nephropathy (IgAN) diagnosis. (**a**) In a patient with IgAN without celiac disease, the IgA is only found in the glomerular mesangium. No colocalization of IgA and tTG is detected; (**b**) representative figure demonstrating colocalization of IgA and tTG (yellow) in the extracellular matrix around the renal tubuli in patients with both IgAN and celiac disease; (**c**) IgA; (**d**) tTG staining in the same specimen. Magnification 20x.

**Figure 2:**
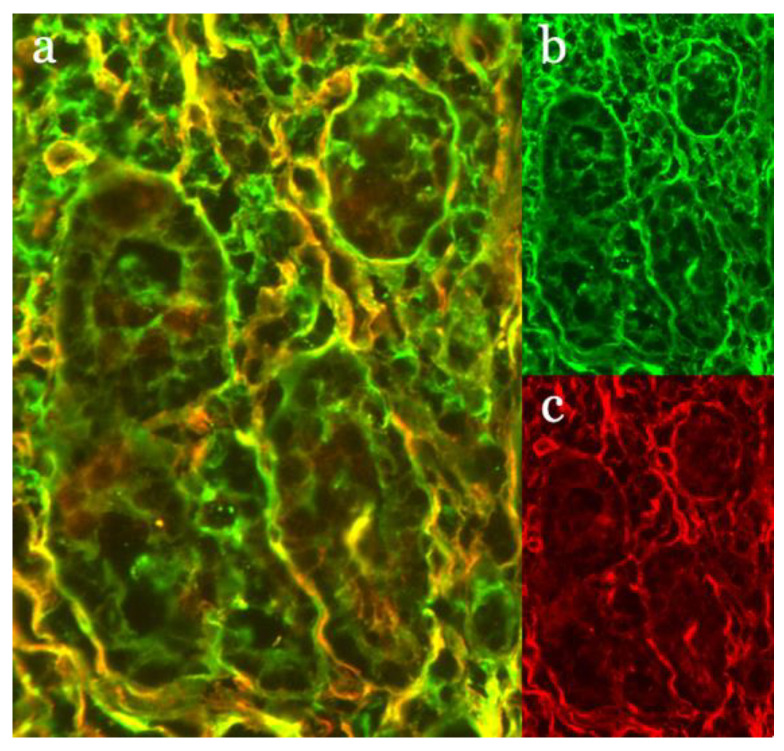
Small bowel mucosal biopsy of a celiac disease patient at the time of diagnosis of celiac disease stained for celiac disease-type IgA-tTG deposits. (**a**) Immunofluorescent staining demonstrating the colocalization of IgA and tTG (yellow); (**b**) staining for IgA (green), and (**c**) tissue transglutaminase (tTG) (red) in the same specimen. Magnification 20x.

By definition, all nine IgAN patients in our case series had glomerular IgA deposits characteristic of IgAN (Figure 1a). Four of the subjects were female (Table 1). At the time of the kidney biopsy and IgAN diagnosis their median age was 34 years (range 20–50 years) and eight of them were on a normal gluten-containing diet (Table 1). One patient (9M) with previously diagnosed celiac disease was on GFD. One patient (1F) was diagnosed with celiac disease during the same treatment episode as the diagnosis of IgAN. During follow-up two additional subjects (2M and 3M) received celiac disease diagnoses after eight and ten years respectively. At the time of celiac disease diagnoses, both of these cases had tTG-targeted autoantibodies in serum and IgA-tTG deposits in the small bowel mucosal biopsies (Figure 2). 

In the kidney biopsies, celiac disease-type deposits characterized by colocalization of IgA and tTG were detected in all three patients with both IgAN and celiac disease on a normal gluten-containing diet (Figure 1b–d, Table 1). It is noteworthy that in two patients (2M and 3M) diagnosed with celiac disease during follow-up, the celiac disease-type IgA-tTG autoantibody deposits were already present in the renal tissue at the time of the kidney biopsies taken eight and ten years prior to celiac disease diagnosis. In all three patients with both IgAN and celiac disease, connective tissue IgA deposits were found around both proximal and distal tubuli. Moreover, in two of these patients (1F and 3M) deposits were also seen in the periglomerular region around Bowman’s capsule. Interestingly, tTG autoantibody levels in the serum taken at the time of kidney biopsy and determined retrospectively from stored samples were already elevated in all these celiac disease patients, including those subsequently diagnosed with celiac disease during follow-up (Table 1). 

Of the five IgAN patients without celiac disease, three had celiac disease-type IgA-tTG deposits around the proximal and distal tubuli in the kidney (4F, 5F, 6M) without having elevated levels of serum tTG autoantibodies at the cross-sectional serologic evaluation at diagnosis (Table 1). Of the remaining two IgAN patients without celiac disease (7F, 8M), neither had celiac disease-type IgA-tTG deposits in the kidney. Of these patients, 7F had no serum tTG autoantibodies, while 8M had no serum sample available for analysis. Patient 9M on GFD due to earlier diagnosed celiac disease, had no celiac disease-type tTG-targeted IgA deposits in the diagnostic kidney biopsy, although serum tTG autoantibody levels were still slightly elevated.

The median follow-up for all patients was 28 (range 5–35) years (mean 23, standard deviation 11 for comparison). Four patients with IgAN and celiac disease were followed-up for a median 28 years (range 5–35) (mean 24, standard deviation 13 for comparison). Two of these had died and two suffered from severe loss of kidney function (eGFR 17 and 23 respectively). Five IgAN patients without celiac disease were followed-up for a median 20 years (range 10–35) (mean 22, standard deviation 11 for comparison). One had normal kidney function, two mild loss of kidney function, and two moderate renal impairment (Table 1). None of these patients required dialysis treatment. No follow-up data on serum tTG autoantibody levels were available. 

## 4. Discussion

In this case series, we found that, during ingestion of gluten, celiac disease-type tTG-targeted IgA deposits were present in the kidney biopsies of all patients with both IgAN and celiac disease and thus our results are in line with the case report by Costa et al. [23]. Two of the IgAN patients with celiac disease (2M, 3M) were found to have IgA-tTG deposits in the kidney years before the diagnosis of celiac disease. Parallel deposits were also detected in the small bowel mucosal biopsies of these two patients (2M, 3M) at the time of celiac disease diagnosis, but unfortunately no small bowel mucosal biopsies taken at the time of IgAN diagnosis were available for the determination of IgA-tTG deposits. However, as small bowel mucosal IgA-tTG deposits may be present prior to small bowel mucosal damage diagnostic for celiac disease [4], the finding of celiac disease-type IgA-tTG deposits in the kidney is interesting and suggests that such deposits may also occur prior to celiac disease diagnosis. It must be noted, however, that both patients (2M, 3M) with IgA-tTG deposits in the kidney also had tTG autoantibodies in serum at the time of IgAN diagnosis. This would suggest that they already had celiac disease at this point, even though the clinical diagnosis of celiac disease came only later. In fact, one of these patients (3M) had tTG autoantibody levels 10 times above the cut-off and this would have been sufficient to warrant a diagnosis of celiac disease under the current guidelines [24]. In any case, as patient 9M with diagnosed celiac disease and already on a GFD at the time of kidney biopsy did not have celiac disease-type IgA-tTG deposits in the kidney, this would suggest that these deposits may be gluten-dependent. The fact that this patient had circulating tTG autoantibodies in low concentrations casts doubt on the strictness of the diet in this patient but may also suggest that these celiac disease-type deposits may disappear from the extraintestinal organs even before the complete clearance of autoantibodies from circulation. 

In our study the IgA-tTG deposits in the kidney were not specific to celiac disease patients since they were also found in three non-celiac IgAN patients without serum tTG autoantibodies. Although no data is available on the presence of extraintestinal IgA-tTG deposits in seronegative celiac disease, such patients have been reported to have such deposits in the small bowel mucosa [6]. Unfortunately, no small bowel mucosal specimens taken at the time of IgAN diagnosis were available for determination of mucosal morphology and IgA-tTG deposits, and therefore we cannot be certain whether these IgAN patients with IgA-tTG deposits in the kidney were indeed antibody-negative celiac disease patients. Moreover, serum samples or small bowel mucosal biopsies during follow-up were not collected and thus it cannot be ascertained whether these individuals developed celiac disease later. However, it is interesting that our earlier studies describe small bowel inflammation and stress in IgAN in the absence of celiac disease [25,26]. Furthermore, increased immune reactivity to dietary antigens, including gluten, has been suggested among IgAN patients even without overt dietary intolerance [20,27]. Intestinal IgA-tTG deposits have been reported in patients without celiac disease and also in a patient with gluten ataxia, in whom the deposits were also found in the brain [7,28]. Hence the renal IgA-tTG deposits in IgAN patients without celiac disease could indicate a similar phenomenon. It is possible that the colocalization of IgA-tTG deposits in the kidney among IgAN patients with celiac disease is a familiar antigen-antibody interaction, while that among patients with IgAN reflects another type of molecular phenomenon related, for instance, to increased expression of tTG in the renal biopsies of IgAN patients [16]. 

In our study the patients with IgAN and celiac disease seemed to have worse outcomes of IgAN than did the patients with IgAN only. This finding is interesting in the light of our earlier study and the Swedish study by Rehnberg and co-workers showing that prognosis in IgAN may be poorer with concomitant comorbid bowel disease [29,30]. However, given the small number of patients and the fact that several factors affected the outcome of IgAN patients [31], conclusions concerning the renal survival of IgAN patients with celiac disease cannot be drawn on the basis of our data. Additionally, the follow-up of IgAN patients with celiac disease was longer, which may likewise affect this finding. In any case, the impact of the celiac disease-type IgA-tTG deposits in the kidney seemed not to be related to renal function or outcome, as also suggested by earlier findings of IgA in the kidneys of celiac disease patients without any renal problems [32]. 

To conclude, tTG-targeted IgA deposits were found in the kidney biopsies of gluten-consuming IgAN patients with or without known celiac disease. The significance of this interesting finding remains open and therefore in the future larger studies, preferably with more data on small bowel histochemistry and regular follow-up serology for tTG antibodies, are needed to investigate the association between celiac disease and IgAN in greater detail.

## Figures and Tables

**Table 1:** Background and follow-up data and biopsy findings among nine patients with IgA nephropathy (IgAN).

		At IgAN Diagnosis				At CD Diagnosis				
Patient/Sex	Diagnosis	Age	IgA-tTG Deposits in the Kidney	Serum tTG Autoantibody Levels (U/mL)		Age	Gastrointestinal Symptoms and Signs	Duodenal IgA-tTG Deposits	Follow-Up ^1^, Years	Disease Progression
	On gluten containing diet									
1F	IgAN + CD	28	Yes	>100		28	Malabsorption	No data	35	eGFR 17
2M	IgAN + CD	35	Yes	36		42	Diarrhea, malabsorption	Yes	28	Dialysis and death
3M	IgAN + CD	41	Yes	>100		51	No symptoms ^2^	Yes	29	eGFR 23
4F	IgAN	20	Yes	1.8		-	-		20	eGFR 110
5F	IgAN	50	Yes	1.4		-	-		35	eGFR 43
6M	IgAN	32	Yes	0.9		-	-		15	eGFR 71
7F	IgAN	28	No	0.7		-	-		10	eGFR 82
8M	IgAN	39	No	No data		-	-		32	eGFR 58
	On a gluten-free diet									
9M	IgAN + CD ^3^	34	No	17		<34 ^4^	No data	No data	5	Death

IgAN, IgA nephropathy; CD, celiac disease; tTG, tissue transglutaminase; eGFR, estimated glomerular filtration rate. tTG autoantibody levels higher than 7.0 U/mL were regarded as positive. eGFR ≥ 90 indicates normal kidney function, eGFR 60–89 mild loss of kidney function, eGFR 30–59 moderate renal impairment, eGFR 15–29 severe renal impairment and eGFR < 15 renal failure. ^1^ Started from kidney biopsy. ^2^ Risk-group screening. ^3^ The diagnosis of CD was made before the diagnosis of IgAN and the patient followed gluten-free diet at the time of kidney biopsy. Initial serum tTG autoantibody level was not known. ^4^ Exact time of diagnosis of CD was not known.

## Data Availability

The data are not publicly available due to ethical and privacy reasons.
